# FeCo/Graphite Nanocrystals for Multi-Modality Imaging of Experimental Vascular Inflammation

**DOI:** 10.1371/journal.pone.0014523

**Published:** 2011-01-14

**Authors:** Hisanori Kosuge, Sarah P. Sherlock, Toshiro Kitagawa, Masahiro Terashima, Joëlle K. Barral, Dwight G. Nishimura, Hongjie Dai, Michael V. McConnell

**Affiliations:** 1 Division of Cardiovascular Medicine, Stanford University, Stanford, California, United States of America; 2 Department of Chemistry, Stanford University, Stanford, California, United States of America; 3 Electrical Engineering, Stanford University, Stanford, California, United States of America; Innsbruck Medical University, Austria

## Abstract

**Background:**

FeCo/graphitic-carbon nanocrystals (FeCo/GC) are biocompatible, high-relaxivity, multi-functional nanoparticles. Macrophages represent important cellular imaging targets for assessing vascular inflammation. We evaluated FeCo/GC for vascular macrophage uptake and imaging *in vivo* using fluorescence and MRI.

**Methods and Results:**

Hyperlipidemic and diabetic mice underwent carotid ligation to produce a macrophage-rich vascular lesion. *In situ* and *ex vivo* fluorescence imaging were performed at 48 hours after intravenous injection of FeCo/GC conjugated to Cy5.5 (n = 8, 8 nmol of Cy5.5/mouse). Significant fluorescence signal from FeCo/GC-Cy5.5 was present in the ligated left carotid arteries, but not in the control (non-ligated) right carotid arteries or sham-operated carotid arteries (p = 0.03 for ligated vs. non-ligated). Serial *in vivo* 3T MRI was performed at 48 and 72 hours after intravenous FeCo/GC (n = 6, 270 µg Fe/mouse). Significant T2* signal loss from FeCo/GC was seen in ligated left carotid arteries, not in non-ligated controls (p = 0.03). Immunofluorescence staining showed colocalization of FeCo/GC and macrophages in ligated carotid arteries.

**Conclusions:**

FeCo/GC accumulates in vascular macrophages *in vivo*, allowing fluorescence and MR imaging. This multi-functional high-relaxivity nanoparticle platform provides a promising approach for cellular imaging of vascular inflammation.

## Introduction

Inflammation is a major contributor to atherosclerosis, a leading cause of death worldwide [1]–[3]. Macrophages in the vessel wall have been associated with atherosclerotic plaque rupture and acute myocardial infarction [4]–[7]. Thus, imaging of vascular macrophages may be useful for characterizing plaque biological activity.

Cellular and molecular imaging of atherosclerosis and vascular inflammation has made significant progress, but remains challenging [8]–[22]. FeCo/graphitic-carbon nanocrystals (FeCo/GC) are composed of an iron-cobalt (FeCo) core with a graphitic-carbon shell [23]. We have shown that the FeCo core provides higher relaxivity for MRI compared to other commercially available contrast agents, while the graphite shell enables biocompatibility and high optical absorbance in the near-infrared region for potential thermal therapy [23]. We have also shown the potential of FeCo/GC for stem cell imaging *in vitro* as well as high-resolution vascular angiography *in vivo* [23], [24], but *in vivo* cellular imaging of FeCo/GC has not been studied.

The aim of the current study was to investigate FeCo/GC for *in vivo* macrophage uptake and imaging in a murine model of vascular inflammation. We show carotid artery macrophages take up FeCo/GC and can be imaged by *in situ* fluorescence and *in vivo* high-field MRI.

## Results

### Fluorescence imaging of carotid arteries after administration of FeCo/GC

**Figure 1:**
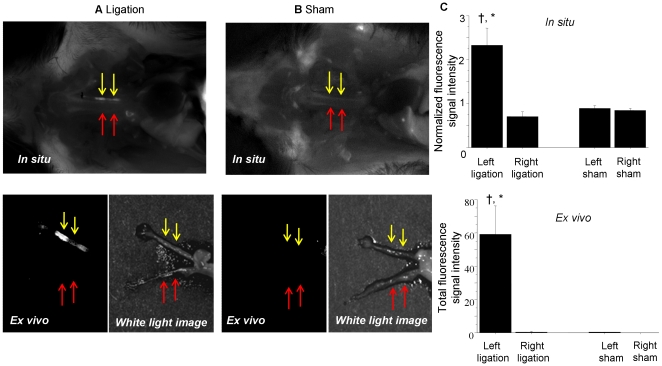
Fluorescence imaging of FeCo/GC in mouse carotid arteries. (**A**) Both *in situ* and *ex vivo* images at 48 hours after FeCo/GC-Cy5.5 injection showed enhanced fluorescence signal localized to the ligated left carotid artery (yellow arrows), but not to the contralateral non-ligated right carotid (red arrows). (**B**) Sham-operated mice showed no significant signal in either the left or right carotid arteries. (**C**) Quantitative analysis of both *in situ* and *ex vivo* fluorescence signal intensity showed significantly greater fluorescence from ligated left carotid arteries compared to non-ligated right carotid arteries. †p = 0.03 vs. right carotid, *p = 0.046 vs. sham left carotid.

Both *in situ* and *ex vivo* fluorescence imaging at 48 hours showed high signal from the ligated left carotid arteries, confirming FeCo/GC uptake in the vascular lesions (Figure 1A; yellow arrows). In contrast, there was no significant FeCo/GC accumulation seen in the non-ligated right carotid arteries (Figure 1A, red arrows). Furthermore, sham operated mice did not show significant signal in either left or right carotid arteries (Figure 1B).

Quantitative analysis of both *in situ* and *ex vivo* signal intensities showed that the ligated left carotid arteries had significantly higher fluorescence than the control (non-ligated) right carotid arteries (Figure 1C; *in situ* (left vs. right): 2.3±0.4 vs. 0.7±0.1, p = 0.03, *ex vivo* (left vs. right): 59.2±17.2 vs. 0.3±0.3, p = 0.03). Also, the ligated left carotids had significantly higher fluorescence than sham left carotids (*in situ* (ligated vs. sham): 2.3±0.4 vs. 0.9±0.1, p = 0.046, *ex vivo* (ligated vs. sham): 59.2±17.2 vs. 0.3±0.3, p = 0.046).

### *In vivo* MRI of carotid arteries after administration of FeCo/GC

**Figure 2:**
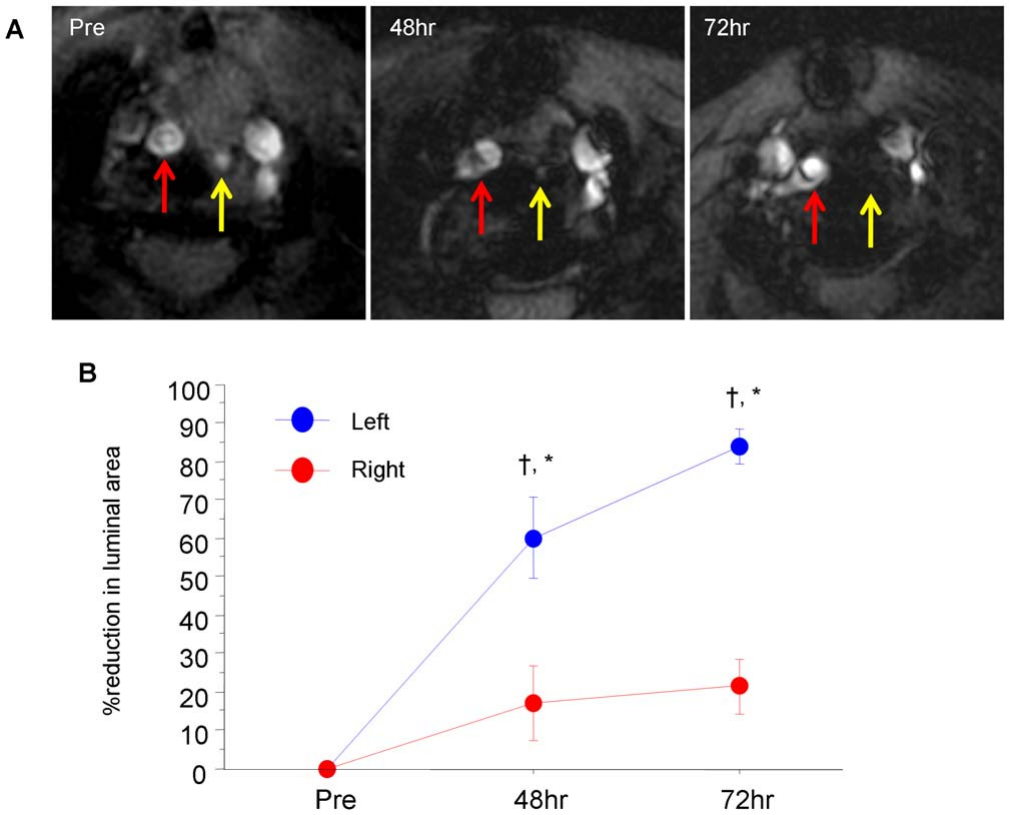
Serial *in vivo* MRI of carotid arteries with FeCo/GC. (**A**) The ligated left carotid artery (yellow arrow), as expected, was smaller than the non-ligated right carotid artery (red arrow) prior to FeCo/GC injection (Pre). After FeCo/GC injection, T2* signal loss of the ligated left carotid artery was seen at 48 and 72 hours. Luminal area reduction was not seen in the control (non-ligated) right carotid artery. (**B**) Quantitative analysis showed significant % reduction in lumen area of ligated left vs. non-ligated right carotid arteries. †p = 0.03 vs. Pre, *p = 0.03 vs. right carotid.

MRI prior to FeCo/GC administration (Figure 2A-Pre) showed that the ligated left carotid artery (yellow arrow), as expected, was smaller than the non-ligated right carotid artery (red arrow). Post FeCo/GC injection, serial MRI showed reduction in left carotid lumen area due to T2* signal loss from FeCo/GC accumulation at both 48 and 72 hours. In contrast, luminal area reduction due to T2* signal loss was not seen in the control (non-ligated) right carotid arteries (Figure 2A).

The measured % reduction of carotid lumen area at 48 and 72 hours for the ligated left carotid arteries was significantly greater than for the non-ligated controls, indicating the accumulation of FeCo/GC in the carotid lesion (Figure 2B; 48 hours (left vs. right): 60.1±10.7% vs. 16.9±9.9%, p = 0.03; 72 hours (left vs. right): 83.9±4.6% vs. 21.3±7.2%, p = 0.03). Furthermore, preliminary testing (n = 3) of an eight-fold lower FeCo/GC dose at higher field strength (7T) also showed T2*-induced lumen reduction in ligated left vs. right carotid arteries (28.0±5.7% vs. −5.56±1.2%, p = 0.1).

### Histology

**Figure 3:**
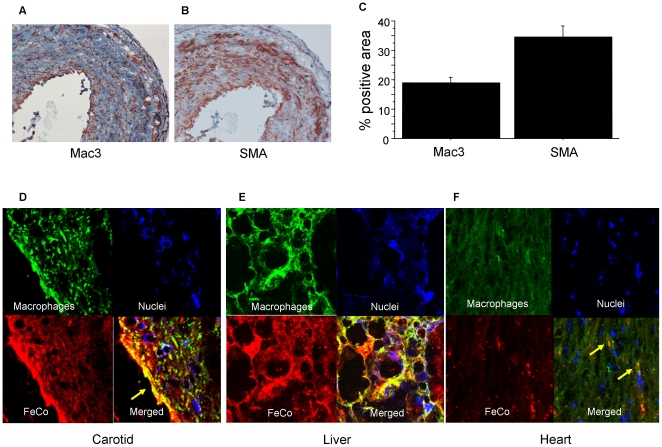
Immunostaining of macrophages and FeCo/GC. (**A**) Immunohistochemical staining showed macrophages (by anti-Mac3 antibody) infiltrating the neointima of the ligated left carotid artery. (**B**) Immunohistochemical staining for smooth muscle cells (by anti-SMA antibody). (**C**) Quantitative analysis of proportion of neointima composed of macrophages and smooth muscle cells. (**D**) Immunofluorescent staining demonstrated macrophages (green), FeCo/GC-Cy5.5 (red), nuclei (blue), and combined staining with strong colocalization (yellow), particularly near the luminal border (arrow). (**E**) Immunofluorescent staining of the macrophage-rich liver also demonstrated strong colocalization (yellow) of FeCo/GC and macrophages. **(F)** By contrast, the macrophage-poor heart tissue showed scant FeCo/GC uptake, with the few spots of uptake colocalizing with macrophages (arrows).

Immunohistochemistry for macrophages demonstrated that there was substantial macrophage infiltration in the neointima of ligated left carotid arteries, accounting for 18.9±1.9% of the neointimal area (Figure 3A,C). There was also significant smooth muscle cell proliferation, accounting for 34.5±3.7% of the neointimal area (Figure 3B,C). Furthermore, carotid immunofluorescence staining showed that FeCo/GC colocalized with macrophages (Figure 3D). The specificity of FeCo/GC for macrophages was further confirmed by immunofluorescence staining showing abundant uptake in the macrophage-rich liver and scant uptake in the macrophage-poor heart (Figure 3E,F).

## Discussion

In an experimental model of vascular inflammation, we have shown that a FeCo-based, graphite-coated nanoparticle can accumulate *in vivo* in vascular macrophages and can be imaged by fluorescence and MRI. To the best of our knowledge, this study is the first to show the use of FeCo or graphite-coated nanoparticles for *in vivo* macrophage imaging. We have previously reported that FeCo/GC exhibited higher *T*1 and *T*2 relaxivities than commercially available iron-oxide- and gadolinium-based contrast agents, with cell hyperthermia capabilities, and without evidence of *in vitro* or *in vivo* toxicity [23], [24]. Furthermore, we have shown that FeCo/GC has a long circulation time and can allow very high-resolution MR angiography (down to 100 µm) [24]. By demonstrating *in vivo* vascular macrophage uptake and imaging, we show that the potent MRI characteristics and potential therapeutic capabilities of FeCo/GC can be applied more broadly to atherosclerosis diagnosis and therapy – from high-resolution angiography to detection of vascular inflammation to macrophage thermal ablation.

Vascular inflammation has been studied by a variety of imaging modalities, including optical, nuclear, x-ray computed tomography, and MRI [8]–[22]. Prior optical studies have used bioluminescence, magneto-optical particles, or enzymatically activated near-infrared agents [8], [12], [14], [17], [21]. MRI studies have primarily used ultra-small superparamagnetic iron-oxide agents for detecting macrophage infiltration in vascular lesions in both experimental and clinical studies [15]–[17], [22] Gadolinium-based micelles and lipoproteins have also been used for macrophage MRI [9], [19]. One study used a combined carbon nanotube/iron-oxide complex for macrophage cell imaging *in vitro* [20].

In addition to the advantageous MRI properties of FeCo/GC discussed above, FeCo/GC can be conjugated with targeting ligands or therapeutic drugs. We have also shown that the graphite shell provides high optical absorbance for photothermal therapy [23], while the FeCo component has the potential for magnetic hyperthermia [25]. Thus, FeCo/GC nanoparticles have a wide range of imaging, targeting, and therapeutic capabilities and warrant further study.

While the carotid ligation model is advantageous in providing a discrete, macrophage-rich vascular lesion, it cannot replicate the complex, chronic plaques that develop in humans. Fluorescence imaging required *in situ* carotid exposure due to limitations in fluorescence tissue penetration. Fully noninvasive fluorescence imaging may be enabled with alternative techniques, such as fluorescence molecular tomography [8], [14]. The MRI approach is likely made more challenging by using a carotid ligation model, as the ligation decreases both lumen size and flow. Aortic imaging in a transgenic hyperlipidemic mouse model is an alternative [8]. Finally, while preliminary studies have shown no acute or chronic toxicity of FeCo/GC [24], more extensive, large-animal toxicity studies are needed prior to clinical translation.

In conclusion, the present study provides evidence that FeCo/GC nanoparticles are promising multi-modality contrast agents for vascular macrophage imaging. Further studies of this multi-functional nanomaterial may enable improved imaging, and therapy, of vascular inflammation in patients.

## Materials and Methods

### Ethics statement

This study was carried out in strict accordance with the recommendations in the Guide for the Care and Use of Laboratory Animals of the National Institutes of Health. The protocol was approved by the Administrative Panel on Laboratory Animal Care at Stanford University (Assurance # A3213-01). All procedures were performed under isoflurane anesthesia, and all efforts were made to minimize suffering.

### Animals

A macrophage-rich murine carotid ligation model was used [21], [22]. Eight-week-old male FVB mice (n = 17) were fed high fat diet containing 40% kcal fat, 1.25% (by weight) cholesterol and 0.5% (by weight) sodium cholate (D12109, Research Diets, Inc. New Brunswick, NJ, USA) [26]. After 1 month on the diet, diabetes was induced by 5 daily intraperitoneal injections of streptozotocin (STZ, 40 mg/kg, Sigma-Aldrich, Saint Louis, MO, USA) dissolved in a citrate buffer (pH 4.5, Sigma-Aldrich, USA) [27]. At day 5 of the STZ injections, serum glucose was measured from tail vein blood using a glucometer. If the glucose level was below 200 mg/dL, animals were injected with STZ for 3 additional days. Two weeks after the initiation of STZ injection, the left common carotid artery was ligated below the bifurcation with the use of 5-0 silk ligature (Ethicon) under 2% inhaled isoflurane [28]. Sham operation was performed by passing the suture under the left carotid artery without constricting the artery. The wound was closed by suture and the animals were allowed to recover on a warming blanket.

### FeCo/GC synthesis

The 7 nm FeCo/GC were synthesized as previously described [23], [24], then sonicated with 1 mg/mL phospholipid-polyethylene glycol (PL-PEG) in water for one hour. To obtain singly suspended FeCo/GC, the solution was centrifuged for 6 hours at 24,000× g. Excess PL-PEG was removed by filtration and UV-vis was used to determine nanocrystal and metal concentration.

For fluorescence imaging experiments the PL-PEG used was DSPE-PEG_5000_-NH_2_ (NOF corp.). For fluorescent dye attachment, Cy5.5-NHS ester was mixed with FeCo/GCPL-PEG-NH_2_ in phosphate buffered saline. Dye that was not attached to the nanocrystal was removed by filtration. Samples prepared for *in vivo* MRI imaging at 3 T were functionalized using DSPE-mPEG_5000_ (1,2-distearoyl-sn-glycero-3-phosphoethanolamine–N-[methoxy(polyethylene glycol)5000], Laysan Bio).

### *In vivo* uptake and fluorescence imaging

To verify *in vivo* uptake of Fe/Co-GC in the carotid macrophages, Cy5.5 was conjugated to FeCo/GC, allowing fluorescence imaging and microscopy. FeCo/GC-Cy5.5 (8 nmol of Cy5.5/mouse; 32.14 µg Fe/mouse) was injected intravenously via tail vein into mice two weeks after carotid ligation (n = 6) or sham operation (n = 2). These mice were imaged at 48 hours using the Maestro™ *in-vivo* imaging system (Cri, Woburn, MA). Under inhalational anesthesia (2% isoflurane), left and right carotid arteries were surgically exposed and *in situ* florescence imaging was performed. Then, the carotid arteries were carefully removed en bloc followed by *ex vivo* fluorescence imaging and immunohistochemistry (see below).

### In vivo MRI

To study noninvasive imaging of FeCo/GC by MRI, ligated mice (n = 6) were injected with FeCo/GC (270 µg Fe/mouse) and imaged serially at 48 and 72 hours on a whole-body 3T MRI scanner (Signa HDx, GE Healthcare) with a 50mT/m, 150T/m/s gradient system and a custom 3 cm surface radiofrequency coil. Another group of mice (n = 3) were injected with an 8-fold lower dose of FeCo/GC (32 µg Fe/mouse) and imaged at 48 hours on a small-bore 7T system (30 cm bore magnet, Varian Inc. plus GE “Micro-Signa” environment), with a 9 cm gradient insert (770mT/m, and 2500T/m/s, Resonance Research, Inc.) and a custom 6-cm RF coil. To detect the T2* effects of FeCo/GC, bright-blood images were acquired using gradient echo sequences (3T: repetition time (TR)  = 100 ms, echo time (TE)  = 10 ms, matrix size  = 256×256, slice thickness  = 1.0 mm, field of view (FOV)  = 3 cm, flip angle  = 60°; 7T: same parameters except TR/TE  = 50 ms/4.2 ms, slice thickness  = 0.5 mm). The slice position was matched using the aortic arch as a reference point.

### Image analysis

For *in situ* carotid fluorescence imaging, regions-of-interest (ROIs) were placed on the left and right carotid regions and trachea. Average signal intensity divided by exposure time for left and right carotids was calculated and normalized to the trachea signal. For *ex vivo* carotid imaging, ROIs were placed around the entire left or right carotid artery and total signal intensity divided by exposure time was calculated. For *in vivo* MRI, FeCo/GC accumulation was assessed by measuring the extent of T2*-induced reduction in carotid lumen size, which was calculated as follows: % reduction of carotid lumen area  =  (1-[post-contrast carotid lumen area]/[pre-contrast carotid lumen area]) x 100% [15].

### Histology

After euthanasia, carotid arteries were cut into two 3 mm sections. These sections, as well as liver and heart, were embedded immediately in optimum cutting temperature (OCT) compound (Sakura Finetek USA, Inc., Torrance, CA) and flash frozen in liquid nitrogen. Frozen sections (5 µm) were fixed in acetone for 10 minutes at 4°C [29]. After sections were washed in PBS, they were incubated with anti-mouse Mac-3 antibody (BD Biosciences, San Jose, CA) or anti-mouse alpha smooth muscle actin antibody (Abcam Inc., Cambridge, MA) overnight at 4°C. Sections were then incubated with biotinylated secondary antibodies at room temperature for 30 minutes. Antigen-antibody conjugates were detected with avidin-biotin-horseradish peroxidase complex (Vector Laboratories, Burlingame, CA) according to the manufacturer's instructions. 3-amino-9-ethylcarbazole was used as chromogen. Sections were counterstained with hematoxylin.

Immunofluorescence double staining with confocal microscopy (Zeiss LSM 510, Carl Zeiss AG, Oberkochen, Germany) was performed to examine colocalization of FeCo/GC-Cy5.5 and macrophages. After incubation with anti-mouse Mac-3 antibody overnight at 4°C, sections were stained with Alexa Fluor 488-conjugated anti-rat IgG (Molecular Probes, Eugene, OR) at room temperature for 2 hours. Finally, sections were stained with DAPI (Sigma-Aldrich) to visualize cell nuclei.

### Statistical analysis

All data are expressed as mean±SEM (standard error of the mean). Comparisons of fluorescence and MRI measures between left (ligated) and right (non-ligated) carotid arteries and between timepoints were analyzed by the Wilcoxon signed-rank test. Comparisons between ligated and sham left carotid arteries were analyzed by the Mann-Whitney U test. P<0.05 was considered statistically significant.
